# Testing machine learning of multimodal digital markers for early detection of cognitive impairment in Alzheimer's Disease rhoda

**DOI:** 10.1002/alz70856_107581

**Published:** 2026-01-09

**Authors:** Joseph Geraci, Edward Searls, Bessi Qorri, Kristi Ho, Alexa Burk, Mike Tsay, Christian Cumbaa, Luca Pani, Larry Alphs, Michael L Alosco, Jesse Mez, Rhoda Au

**Affiliations:** ^1^ Department of Pathology and Molecular Medicine, Queen's University, Kingston, ON, Canada; ^2^ Arthur C. Clarke Center for Human Imagination, School of Physical Sciences, University of California, San Diego, La Jolla, CA, USA; ^3^ NetraMark Corp, Toronto, ON, Canada; ^4^ Boston University Chobanian & Avedisian School of Medicine, Boston, MA, USA; ^5^ Department of Psychiatry and Behavioral Sciences, Leonard M. Miller School of Medicine, University of Miami, Coral Gables, FL, USA; ^6^ Department of Biomedical, Metabolic, and Neural Sciences, University of Modena and Reggio Emilia, Modena, Italy; ^7^ Boston University Alzheimer's Disease Research Center, Boston, MA, USA; ^8^ Boston University Chronic Traumatic Encephalopathy Center, Boston, MA, USA; ^9^ Framingham Heart Study, Boston, MA, USA; ^10^ Boston University School of Public Health, Boston, MA, USA; ^11^ Framingham Heart Study, Framingham, MA, USA

## Abstract

**Background:**

Alzheimer's disease (AD) precision medicine will advance through the application of two key technological advances: 1) digital technologies that can more deeply characterize clinically relevant symptoms and 2) machine learning (ML) approaches the can classify subgroups with shared characteristics that could align with specific treatment plants. This study leverages a digital data collection platform for enhanced characterization and NetraAI, an artificial intelligence (AI) platform to analyze multimodal data to differentiate causal and non‐causal subpopulations within a cohort and integrates a “No Call” system to exclude ambiguous data points.

**Method:**

We analyzed data from 98 Boston University Alzheimer's Disease Research Center participants and 453 variables derived from digital tasks administered over two months. Eight participants were clinically diagnosed as mild cognitive impairment. Digital measures included sleep metrics (57 measures), clinical scales (324 measures), and cognitive performance assessments (72 GoNoGo and Code Substitution measures). Of the 98 subjects, 81 were cognitively unimpaired and 17 transitioned to MCI during the course of study enrollment.

**Result:**

Sleep‐derived metrics, including 3% and 4% desaturation thresholds (*p* = 4×10^−5^, *p* = 7×10^−5^), and periodicity (eLFCnb) (*p* = 0.008) characterized one population of 8 participants, 7 of whom had been diagnosed with MCI. Incorporating maximum heart rate, another sleep metric, distinguished another subpopulation of 8 subjects (6/8 were diagnosed MCI) with elevated heart rate (*p* = 10^−10^).

We examined 81 cognitively intact (e.g., non‐transitioners; Class 0) and 17 MCI transitioners (Class 1) related to Go/No‐Go and Code Substitution tasks. Go/No‐Go Inter‐Trial Intervals (ITI), REM sleep percentage, and maximum apnea duration were key predictors. Shorter, more stable ITI times (inter‐trial intervals between tasks), higher REM sleep percentage, and shorter apnea durations were strongly correlated with non‐transitioners. A 10‐fold cross‐validation yielded an average accuracy of 80.89%.

**Conclusion:**

Our findings present an ongoing effort on the potential of explainable AI to validate digital measures to identify those with MCI. While the current model effectively identifies prevalent non‐transitioners, it remains limited in identifying prevalent transitioners. Future research will focus on refining model sensitivity and balancing classification performance.

